# Priority setting at the clinical level: the case of nusinersen and the Norwegian national expert group

**DOI:** 10.1186/s12910-021-00623-5

**Published:** 2021-05-04

**Authors:** Morten Magelssen, Magnhild Rasmussen, Sean Wallace, Reidun Førde

**Affiliations:** 1 Centre for Medical Ethics, Institute of Health and Society, University of Oslo, Pb. 1130 Blindern, 0318 Oslo, Norway; 2 Department of Clinical Neurosciences for Children, Oslo University Hospital, Oslo, Norway; 3 Unit for Congenital and Hereditary Neuromuscular Disorders, Department of Neurology, Oslo University Hospital, Oslo, Norway

**Keywords:** Bedside rationing, Norway, Nusinersen, Priority setting, Rationing, Resource allocation, Spinal muscular atrophy

## Abstract

**Background:**

Nusinersen is one of an increasing number of new, expensive orphan drugs to receive authorization. These drugs strain public healthcare budgets and challenge principles for resource allocation. Nusinersen was introduced in the Norwegian public healthcare system in 2018. A national expert group consisting of physicians was formed to oversee the introduction and continuation of treatment in light of specific start and stop criteria.

**Methods:**

We have studied experiences within the expert group with a special emphasis on their application of the start and stop criteria, rationing of treatment, and experienced moral dilemmas. A research interview with six members of the national expert group was performed, then analysed with manifest content analysis. The analysis was supplemented with publically available sources on priority setting and the process leading up to the introduction of nusinersen and the establishment of the expert group.

**Results:**

Sixty-six patients have received treatment within the first 25 months since the national expert group’s establishment. Treatment has not been discontinued for any patient. No patients under 18 years of age have been denied treatment, as those who were referred at this age were all deemed to fulfill the start criteria. The expert group has, however, increased geographical treatment equity and facilitated important cooperation at the national level. Furthermore, it has enhanced open and critical discussions of both medical issues and new ethical dilemmas.

**Conclusion:**

Although facilitating equal access to treatment for SMA patients, the national expert group has not discontinued treatment for any patient. It is suggested that in order for clinicians to be able to ration care for individual patients, they require both adequate support and sufficient formal authority. Start and stop criteria need to be re-evaluated as more knowledge and experience are gained regarding the treatment.

**Supplementary Information:**

The online version contains supplementary material available at 10.1186/s12910-021-00623-5.

## Background

Resource allocation and prioritizing within healthcare systems is complicated and has medical, economic and ethical implications. Due to the rising costs associated with modern diagnostic and treatment technologies, there is an increasing need for rational allocation of resources to reconcile finite health budgets with just and optimal treatment strategies [1].

The major focus of policy-makers’ and academics’ attention has been priority setting theory and national guidelines at the *macro* (national, political) level. Much less attention has been given to rationing at the *meso* (institutional) and, especially, the *micro* (clinical) level; whether rationing at the micro/clinical level is possible or desirable, and how clinicians should be aided in this task [2–4]. For clinicians, making rationing decisions for individual patients is often considered difficult. It has been claimed that this is partly so because rationing involves modes of deliberation alien and perhaps harmful to the ethics of the physician–patient relationship [3]. In due course, it will become clear how the present case concerns clinical/bedside rationing.

The high costs and increasing availability of new, disease-specific drugs for rare diseases—orphan drugs—is the cause of considerable ongoing debate, both at national levels and internationally. Since 2015 almost half of the drugs approved by the FDA have been orphan drugs, and these cost on average roughly five times as much as other medications [1, 5]. Standard methods of assessing cost-effectiveness of orphan drugs are often not applicable due in part to the rarity and severity of the disease and lack of long-term data on the effects of treatment [1, 6, 7].

### Priority setting in Norway

Norwegian health care is, by and large, publicly funded, and Norway spends more money on health care than most other countries [8]. Five governmental commissions on resource allocation have been appointed between 1985 and 2018. A 2016 government white paper reaffirmed that prioritization of healthcare should be based on three criteria which should be considered together: the utility (health benefit) of the service; the resource use of the service; and the severity of the condition [9]. These criteria have since been enshrined in law.

In 2013, The National System for Managed Introduction of New Health Technologies within the Specialist Health Service was launched. As a central part of the system, «Beslutningsforum» (literally “Decision forum”), a board consisting of the directors of the four regional health care trusts and a patient representative, has the final say in whether new methods should be accepted into the public healthcare system.

The Norwegian resource allocation system is in particular challenged when new drugs promise treatment for severe conditions where no previous effective treatment has been available [1]. Nusinersen (Spinraza®), a new drug for patients with spinal muscular atrophy (SMA), is an example of this [7, 10]. We describe the Norwegian attempts to offer this expensive treatment and how expert clinicians close to this patient group have identified and handled the dilemmas the treatment has given rise to.

### Spinal muscular atrophy (SMA)

SMA is a rare genetic disorder that causes progressive muscle atrophy and weakness with loss of movement, respiratory failure and feeding difficulties. It is most commonly caused by mutations in the survival motor neuron 1 (SMN1) gene. This results in decreased expression of the survival motor neuron (SMN) protein causing degeneration of motor neurons in the spinal cord and brain stem.

This most common form of SMA is divided into three main types, based on the age of onset of symptoms and the highest motor function the patient has ever attained. The most severe form, SMA type I, presents with clinical symptoms before six months of age and is associated with a median life expectancy of less than two years without implementation of respiratory support. Patients with SMA type II do not obtain independent walking, but most often reach adult age. Onset of SMA type III occurs after 18 months of age when the patient has attained the ability to walk, but motor abilities will be reduced at a later stage.

The treatment offered to SMA type I has until recently been palliative only. At least in the Nordic countries it was deemed that it was wrong to offer invasive ventilatory support because this would not alleviate the progressive motor deficit leading to complete paralysis. For SMA type II and III, multidisciplinary habilitation has been the main treatment approach, including physiotherapy, often scoliosis surgery, and for type II quite often also ventilatory and nutritional support.

### Nusinersen introduced in Norway

Nusinersen is an antisense oligonucleotide that is administered by lumbar puncture. It is given six times the first year, then three times annually thereafter.

When treatment with nusinersen was started in Norway, the documentation of effect was promising, but not substantial and included mainly SMA type I [11]. Shortly afterwards, promising interim results were reported also in patients with later-onset SMA [12].

In Norway, nine children with infantile-onset SMA were included in Biogen’s expanded access program, which lasted until nusinersen was approved by the European Medicines Agency in May 2017. Thereafter negotiations were initiated between Biogen and the Norwegian Medicines Agency; and finally in February 2018, “Beslutningsforum” accepted to reimburse nusinersen treatment in children up to 18 years of age on the condition that they fulfilled certain additional criteria regarding genotype, age at onset and ventilatory status (Table 1).

**Table 1:** Summary of main start and stop criteria for nusinersen treatment in Norway [13]

**Start criteria**
Minimum two copies of the SMN2 gene
Patient should not be dependent on assisted ventilation or extra oxygen to keep an SaO2 > 95%
Regarding SMA 3, patients with onset before three years of age may in certain situations be eligible for treatment
**Stop criteria**
SMA 1: Reduction in score on defined motor tests or deterioration regarding nutritional and respiratory status despite treatment
In need of assisted ventilation more than 16 h/day
SMA 2 and 3: Reduction in score on defined motor tests. Deterioration in respiratory status

These criteria were suggested by pediatricians before review by all the regional hospitals’ medical directors. All three start criteria must be fulfilled for treatment to be started. However, the third start criterion is vague and opens up for both narrow and broad interpretations. For the stop criteria, stopping treatment should be considered if one or more were fulfilled.

The exact price for treatment was kept secret, contrary to the Norwegian ideal of openness in priority setting matters, as Biogen demanded secrecy as a condition for a price reduction. However, it was known to all involved that the price of nusinersen was exceptionally high; before the discount was applied, one year’s treatment was priced at approximately € 300,000 per patient. Many patients need hospitalization for the drug to be given by lumbar puncture, and this contributes to the total resource use. Oslo University Hospital has had the responsibility to start up all treatment nationally.

### Establishment of an expert group

As this was a novel and extremely expensive treatment, “Beslutningsforum” considered that expert knowledge was necessary for proper inclusion and exclusion of patients. A national expert group was appointed to ensure that access to nusinersen was equitable, including geographically, and that treatment was medically indicated according to the defined criteria (Table 1) [13]. The criteria served to identify non-responders and to prevent patients from being exposed to invasive and expensive treatment without effect. The group consists of child neurologists from Norway’s four health regions, one representative from the national expert service on long-term mechanical ventilation and one representative from the department of anaesthesiology at Oslo University Hospital, where all the nusinersen treatments are started and where all the patients up to now have been assessed at least once a year. An adult neurologist was later included. The potential role of a professional ethicist in the group was also discussed, but so far none has been included.

This is the first time that “Beslutningsforum” has established a national expert group to oversee treatment; such bodies do not exist for any other diagnosis/treatment constellations. Although the mandate does not mention broader responsibility for taking costs and cost-effectiveness for individual patients into account, it is likely that an important motivation for establishing the expert group was to contribute to keeping total costs down. Priority setting and cost control is a main responsibility for “Beslutningsforum”, and nusinersen was especially controversial in being exceedingly expensive.

The expert group meets (often digitally) three to four times a year. Based on reports from Oslo University Hospital and local hospitals each patient is assessed for the first time 14 months after start of treatment. From the outset it was perceived important that the clinical assessments should be systematic, as far as possible based on objective criteria. Scores on standardized motor tests performed by trained physiotherapists give the main information about the development of motor function. In accordance with the criteria, the treatment is to be discontinued if there is a deterioration in function as defined by the motor score, but respiratory function and to some extent nutritional status are also included in the evaluation.

## Methods

The aim of the study was to describe how the expert group has functioned in its first two years after being established, with special emphasis on the application of start and stop criteria and thus on rationing on the clinical level.

A case study with a tailored design, the data consist of one focus group interview with members of the national expert group in addition to publically available sources on priority setting, nusinersen, and the process leading up to the granting of public funding and the establishment of the expert group. The interview was performed in March 2020 at Oslo University Hospital and lasted 90 min. Six members of the expert group participated in the meeting, of which three via videolink. All six agreed to be interviewed.

The interview was semi-structured with the help of an interview guide detailing the topics to be covered (Additional file 1: Appendix 1). The interview was audiotaped and then transcribed. The transcript was analysed through manifest content analysis and in light of the supplemental data. In a manifest content analysis, the researchers describe what is directly conveyed by the informants [14, 15]. This method is apt when the data material is of limited extent and one wants to capture informants’ own experiences and viewpoints.

In order for the analysis to be sufficiently independent, the interview was performed and analysed by two academic medical ethicists who were independent of the clinicians (MM and RF). The two clinician co-authors (MR and SW) contributed before the interview in introducing some of their key concerns, throughout the interview as informants, and in the revision of the Results and Discussion sections by clearing up misunderstandings, increasing precision and introducing nuances.

## Results

The analysis resulted in four main categories, as shown in Table 2. Twenty-five months after the introduction of nusinersen in Norway’s public health service, 66 patients in total have been treated, most of them having had the diagnosis for several years. Ten of them were included before the start and stop criteria were established, nine of the ten as part of an expanded access program. From having no disease specific treatment available, the novel treatment has given this patient group cause for optimism. The ability to provide effective treatment has also led to a more systematic patient follow-up and assessment, with for instance more aggressive physiotherapy and more focus on respiratory support and nutritional management. This is seen as a positive “side effect” of the introduction of nusinersen.

**Table 2:** Main categories with sub-categories, with example quotes from the interview

Category	Example quote
Rationing treatment?	“The challenge is, in a way, to be able to pick out those who have insufficient effect. And what is sufficient effect? There are these borderlands that are very difficult”
The weakness of “objective” criteria	“These measurements are not perfect, and they are a pseudo-measure, we think, for quality of life, really. Which is very hard to measure”
Members’ overall assessment of the group	“The expert group I think has worked very well, I think it’s a very good model for other similar rare diseases”
New ethical dilemmas	
Quality of life	“But the [SMA] 1’s are the dilemma that we, that I, prevent from dying, and that we often ‘convert’ to poor [SMA] 2’s”
Disagreement	
Resource use	“In any case there are delays, and that [SMA patients] take up a very large part of our department, that’s clearly the feedback from our colleagues, they think it’s far too many Spinrazas [i.e., nusinersen treatments]”

### Rationing treatment?

Based on international incidence data the group believed that all or the vast majority of patients are being referred or at least discussed with Oslo University Hospital. (To their knowledge, two patients with neonatal onset had not been formally referred.)

One patient referred was deemed not to fulfill the start criteria, mainly because of age, and was denied treatment. All other patients who were remitted after reimbursement were accepted, whether newly diagnosed or with long standing disease, because they were with one exception deemed to fulfill the start criteria. The exception was one presymptomatic sibling in whom it was decided to start in accordance with reported better outcome in presymptomatic patients [16]. 54 patients had been evaluated after 14 months of treatment, and 10 had also been evaluated at 26 months.

Notably, treatment has not been discontinued in any patient. The expert group has concluded that no patients have definitively deteriorated. For some of the patients, a thorough discussion was needed to reach this conclusion. Thus, the introduction of start and stop criteria and the national expert group has so far led to only one of the patients referred being denied treatment.

The terms “reduction” and “deterioration” in the stop criteria have been applied first and foremost to the scores on the formal motor tests and mean lower points on the scales. However, the testing physiotherapist would often have commented in situations with a reduced score that the patient for example had been very tired as a consequence of recent travels and poor sleep or had had a recent orthopaedic intervention. In such situations, treatment was allowed to go on for another four months. When the scores next time had improved, the patient was deemed not to have definitely deteriorated. The expert group considered this to be a fair way of using the criteria in a clinical setting. It is also considered in the literature that scores of ± 2 points may be judged as stability [17]. Respiratory function was also taken into account.

The stop criteria turned out to be less objective and practicable than expected and intended. Scores would sometimes not correlate well with the patient’s or the next of kin’s assessment of function and effect. Importantly, the stop criteria did not catch all aspects of the child’s total benefit from treatment confirmed through tests not included in the criteria. The group also felt there was a need to consider “softer”, less objective parameters, such as an increased level of energy as assessed by patient and parents, or increased endurance and greater independence in functioning, for instance preserved or increased dexterity in operating cell phones and wheelchairs.

According to informants the patient’s next of kin would “always” be in favor of continued treatment, apparently placing great hope in the effect of the drug.

The expert group members stated that their role as the patient’s doctor and advocate put them too close to the patient for treatment withdrawal to be (psychologically, professionally) fully comfortable for them without convincing evidence of deterioration. Several claimed that if the expert group in this situation was to agree that a patient’s nusinersen treatment should be discontinued, they might still feel the need for backing from an outside authority—the hospital clinical ethics committee was mentioned—to withdraw treatment. This was because such a decision was felt to be likely to lead to a formal complaint process and/or negatively affect the patient group’s confidence and relationship with the treatment team or lead to media involvement.

Closely linked to this is the group’s lack of emphasis on costs of treatment. Group members stated that considering costs was not an explicit mandate for the group, that they did not feel qualified to do so, that they had had no two-way communication with “Beslutningsforum”, and that it was unknown to them which patient groups might be denied treatment as a consequence of the resources being spent on nusinersen.

### The weakness of “objective” criteria

After new knowledge has been gained from experience with the treatment, the expert group has recommended adjustments to the start and stop criteria, including to supplement the more objective criteria with more soft criteria also based on patients’ and parents’ evaluation of effect. From previous studies it was reported that early initiation of treatment gave the best results [11]. Later reports provide evidence that presymptomatic initiation would be associated with an even better effect [16]. Therefore, the expert group has recommended to include presymptomatic infants.

Experience has also shown that the patients who have had symptoms for a long time can benefit from treatment. According to the start criteria, the treatment could be offered to SMA I and II and in certain situations to type III manifesting before the age of three years. However, for teenagers with type III who had been sick for several years, it could be difficult to decide the exact age of onset. It was therefore suggested to disregard the debut age requirement and instead offer treatment of type III to those who had obvious signs of clinical deterioration, a situation in which it would be easier to evaluate the effect of nusinersen. Additionally, the group commented that some of the criteria concerning respiratory status might be difficult to apply in practice.

Furthermore, the start and stop criteria necessitated tests that were time- and resource-consuming. This was seen as a burden for patients and parents, also emotionally, as something they dreaded long in advance, mainly because the results might indicate stopping the treatment. Parents could sometimes try to influence test scores out of fear that their child would be taken off treatment, for instance by reducing the daily number of hours of ventilation support in the days before testing to “prove” that the child had not deteriorated regarding respiratory function.

### Group members’ overall assessment of the national expert group

The national experts agreed that professional cooperation on a national level has been important and has secured open and critical discussions of medical issues and of difficult ethical dilemmas (see below). The start and stop assessments have been standardized leading to equality of treatment independent of geography and have also increased the quality of the assessments. It is also perceived that *if* limits are to be set for individual patients, this group’s joint conclusion increases legitimacy. All in all, the informants judged the “expert group model” to be helpful, and likely to be so also for other diagnoses within the field of child neurology, for instance, Duchenne muscular dystrophy.

### New ethical dilemmas

The informants pointed out that whereas the new treatment led to genuine and significant gains for the majority of patients, it also gave rise to new ethical dilemmas, many of which were related to priority setting and resource use:

First, whereas nusinersen was of clear benefit to many patients, it also led to the prolongation of lives with extremely pronounced handicap. Some patients still required comprehensive and costly care services. Informants questioned whether nusinersen was truly appropriate use of scarce healthcare resources in all cases; in the case of some patients with uncertain effect, it might be “an expensive placebo”.

Second, informants said that they sometimes observed disagreement between (typically teenage) patients and their parents as to whether treatment should be continued. Typically, parents wanted treatment to go on whereas patients would sometimes emphasize questionable effect and the burdensome treatment which involved hospitalization and lumbar puncture.

Third, increased attention to SMA patients, in connection both with treatment, hospitalizations, and time-consuming clinical testing, would lead to increased waiting lists and less attention to other patient groups, to an extent that was difficult to quantify. In effect, SMA patients were prioritized, possibly at the expense of certain other patients within the child neurology field. However, the possibility to give effective, causal treatment in this progressive neurological disorder was nevertheless perceived as a huge step forward.

Informants thought that the expert group was of significant help in discussing such dilemmas. They also thought that some dilemmas ought to be taken to a hospital clinical ethics committee.

## Discussion

The costs of nusinersen to SMA patients garnered significant attention in Norwegian news media [10]. The “Beslutningsforum” was under some pressure to say “yes” to nusinersen. Doing so would entail breaking with established practices on willingness to pay; due to the very high cost, nusinersen had a much lower cost-effectiveness than other publically financed medications [7]. In addition, at the time of the review, the scientific evidence of the effect of nusinersen was promising but limited with a lack of long-term follow-up data. An examination of this expert group’s work is of significant interest in order to increase understanding of what happens when an expensive, disease modifying treatment is introduced to a seriously ill patient group previously lacking effective or disease specific treatment.

It is interesting to find that after two years the expert group has yet to withdraw nusinersen treatment from a single patient. There are several explanations for this. First, the effect of treatment assessed clinically and subjectively by most patients and parents, has been better than anticipated. Second, in cases with marginal improvement or lack of symptoms of definite deterioration, stopping treatment is professionally, emotionally and perhaps also morally difficult for clinicians, according to members of the expert group. How is the utility (health benefit) criterion to be interpreted in these cases when objective tests according to the criteria do not necessarily indicate stopping treatment, but the patient still has a severe handicap? It can be discussed whether the start criteria have been too wide and whether (mere) stabilization ought always to be sufficient warrant for continuing treatment. A stabilization of a progressive disease will be desirable for the majority of patients. However, in certain cases with SMA there may be stable periods also during the natural course. Finally, it was not in the expert group’s mandate to ration, although the resource allocation criteria were well established in Norway.

### The challenge of saying no at the bedside

Priority setting at the clinical level—rationing at the bedside—is hard. At the core of the problem is the uneasy relationship between “dual obligations” that clinicians are expected to shoulder: On the one hand they are to be stewards of societal resources, allocated to finite healthcare budgets. On the other hand they are to be advocates for their individual patients and the patient groups for which they have special responsibility. Arguably, the traditional ethics of the medical profession emphasizes the latter without giving equal attention to the former. Furthermore, physicians are typically not trained in the concepts and tools of clinical priority setting; the clinicians were unsure of how to include resource concerns in their clinical-ethical reasoning and did not perceive it as their explicit mandate to do so.

The age criterion is an objective and absolute criterion. When nusinersen was introduced only to patients below age 18, this was heavily critized by patient groups as it was seen as unfair and cruel. No patient above 18 years at referral has started treatment.

For physicians to assume responsibility for explicit and consequential rationing decisions, there is a need for sufficient information on the economic impact of the treatment at the micro and macro levels as well as *support* and *authority* for this decision-making process. The expert group was given the formal authority to apply the start and stop criteria. However, the members of the group did not perceive themselves as qualified to ration up against other patient groups, and the group’s mandate to ration care was not explicit. Also, the stop criteria after some time turned out not to be sufficiently comprehensive or practicable for the purposes of setting limits to treatment. In addition, the group’s task was made difficult with the relative lack of robust clinical evidence and the continuously evolving evidence of effect, thus creating a need for continuous re-evaluation of the established criteria. A closer dialogue with “Beslutningsforum” during this process might have been helpful.

It is notable how the informants argued for continued treatment also in cases where health benefits apparently were slight. If one were to estimate the cost per QALY achieved for a group of these patients the cost would likely be sky high.

### Bedside rationing in the future

As we enter the age of precision medicine, public health systems face a deluge of new, very costly drugs and must find a way to make just priorities—whilst neither using up limited budgets nor denying patients efficacious treatment. The study indicates that national clinician expert groups might improve equity and provide a forum for sharing experiences with treatment. Yet it does not indicate that delegating responsibility for rationing to clinician expert groups would be a solution to the challenges of bedside rationing.

One further option would be to give such expert groups more unequivocal criteria and more explicit authority to ration. This could be coupled with more training in the theory and practice of priority setting for the group members. However, clinicians’ primary loyalty would still be with their patients. It is therefore an open question whether such a group with enhanced authority and competence would be able to ration care. Another option is to invest authority in an external actor without the ties that come with clinical responsibility, such as the hospital’s medical director or a clinical ethics committee. However, with the increased distance from the bedside also comes a loss of the clinician’s detailed knowledge about the patient, condition and circumstances which might be relevant for decision-making. A further option is for bodies such as “Beslutningsforum” to turn down new drugs on a stricter assessment of cost-effectiveness. A problem with the latter option is that it is blunt; it does not allow for the *individual*, discretionary assessment of benefit and costs in the case of each patient—which motivated moving these decisions closer to the bedside in the first place.

## Conclusion

The study indicates that throughout its two first years of operation, the national expert group on SMA has been of very limited help in fair resource allocation. Our study also shows how difficult it is to employ start and stop criteria in treatment with insufficient evidence and limited clinical experience. However, to the clinicians taking part the expert group has been valuable for fairness and decision-making in other ways, such as establishing similar practices and allowing for the deliberation on the new dilemmas raised by the treatment. In this respect, the group might constitute an exemplary model for other rare diseases.

## Supplementary Information

**Additional file 1.** Interview guide.

## Data Availability

In order to protect informants’ anonymity, the interview transcript will not be shared. For inquiries about the data the corresponding author can be contacted.

## Abbreviations

SMA: Spinal muscular atrophy
SMN: Survival motor neuron
QALY: Quality-adjusted life year
