# Supplementary material for: Priority setting at the clinical level: the case of nusinersen and the Norwegian national expert group
Source: BMC Med Ethics. 2021 May 4;22:54. doi: 10.1186/s12910-021-00623-5 (PMC8094497; doi:10.1186/s12910-021-00623-5)
Supplement: Supplementary file 1 — Additional file 1. Interview guide. [file 12910_2021_623_MOESM1_ESM.docx]

**Appendix 1.** Interview guide for study on expert group on nusinersen treatment for spinal muscular atrophy (SMA) – translated from Norwegian to English

*Inclusion in treatment*

• How have you experienced the inclusion of patients in terms of fairness and access?

• How to inform in SMA type 1? Is it still acceptable *not* to start treatment even if the inclusion criteria are met?

• Is the absolute age limit fair; can it be defended?

• Have other patient groups in the field of pediatric neurology received delayed / poorer treatment? Which groups, and what consequences has it had? Can this be defended?

*Stop criteria*

• Have any patients been taken off the drug so far? If not, what are the reasons?

• Are the stop criteria good enough, should they be supplemented with other requirements?

• Should effect evaluation be supplemented with effect measures that capture small advances that are nevertheless very important for the patient?

• Has the requirement for objective assessment criteria affected parents and children along the way? If yes, how? Does it affect the relationship of trust between doctor and parents / patient?

• Are the children's experiences taken into account sufficiently?

• What determines in practice whether the treatment is continued?

*About the prioritization work in practice*

• Does the expert group agree on the criteria, or is there a professional disagreement, or how is this resolved?

• Where is the loyalty of the responsible doctor; to the patient or to the overall guidelines?

• Are the members too close to the specific patients? Can you give an example of loyalty conflicts in practice and how this was resolved?

• Are there others who should also have been part of the national expert group?

• Based on the experience so far, how do you evaluate the prioritization and resource use of this drug compared to the treatment of other serious diseases?
